# Supplementary figures and images for: Chikungunya virus requires an intact microtubule network for efficient viral genome delivery
Source: PLoS Negl Trop Dis. 2020 Aug 7;14(8):e0008469. doi: 10.1371/journal.pntd.0008469 (PMC7413472; doi:10.1371/journal.pntd.0008469)

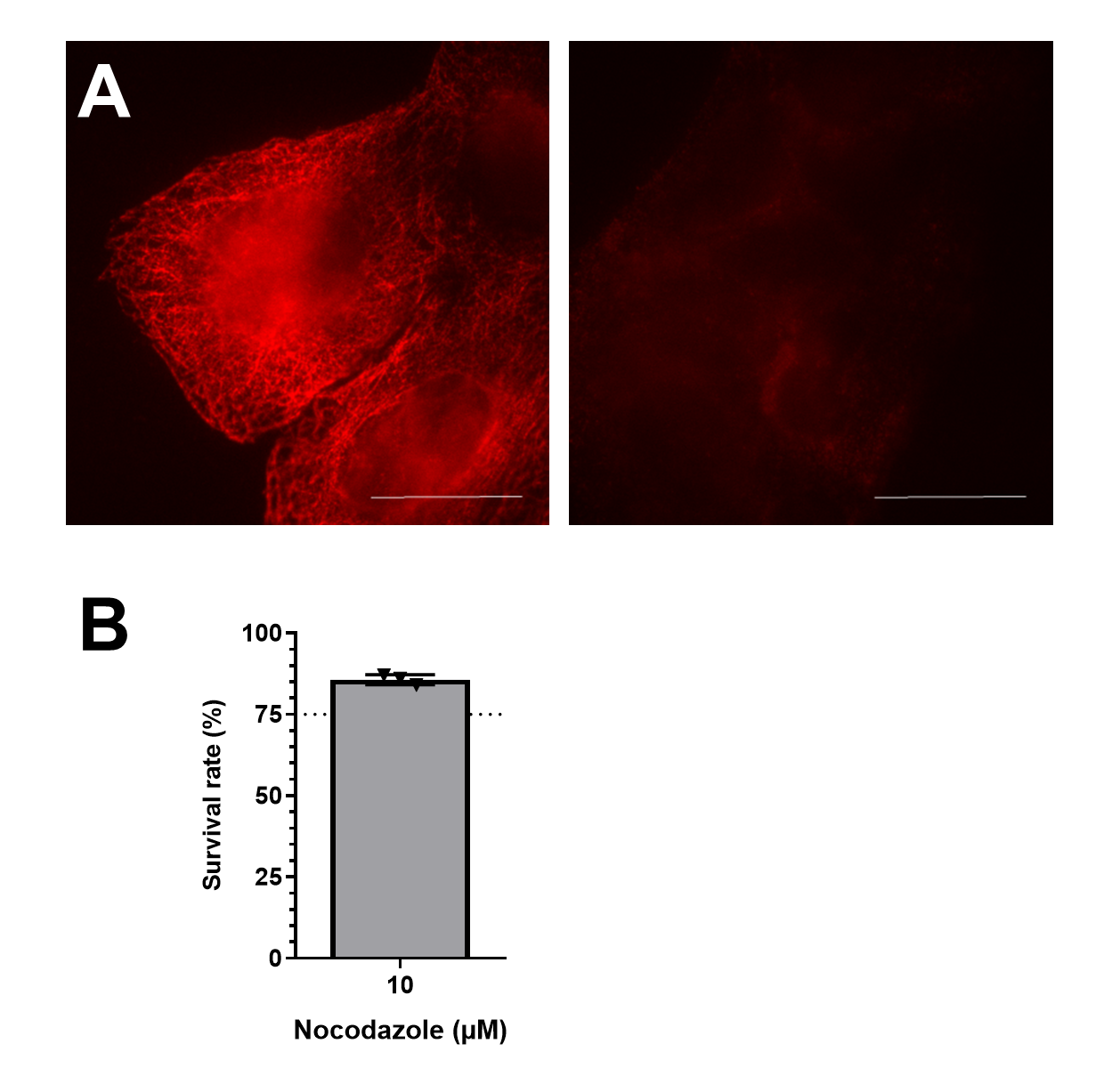

Supplement: S1 Fig — (A) Representative pictures of the α-tubulin staining in U-2 OS cells in absence (left) and presence (right) of 10 μM nocodazole for 2 h. Scale bar: 25 μm. (B) Cell viability of U-2 OS cells upon treatment with nocodazole. U-2 OS cells were treated for 18 h, after which cell viability was assessed using standard ATPlite assay. Dotted line indicates 75% cell survival. A total of three experiments were performed in triplicate. Each dot represents the average of an independent experiment. Error bars represent SD. (TIF) [file pntd.0008469.s001.tif]

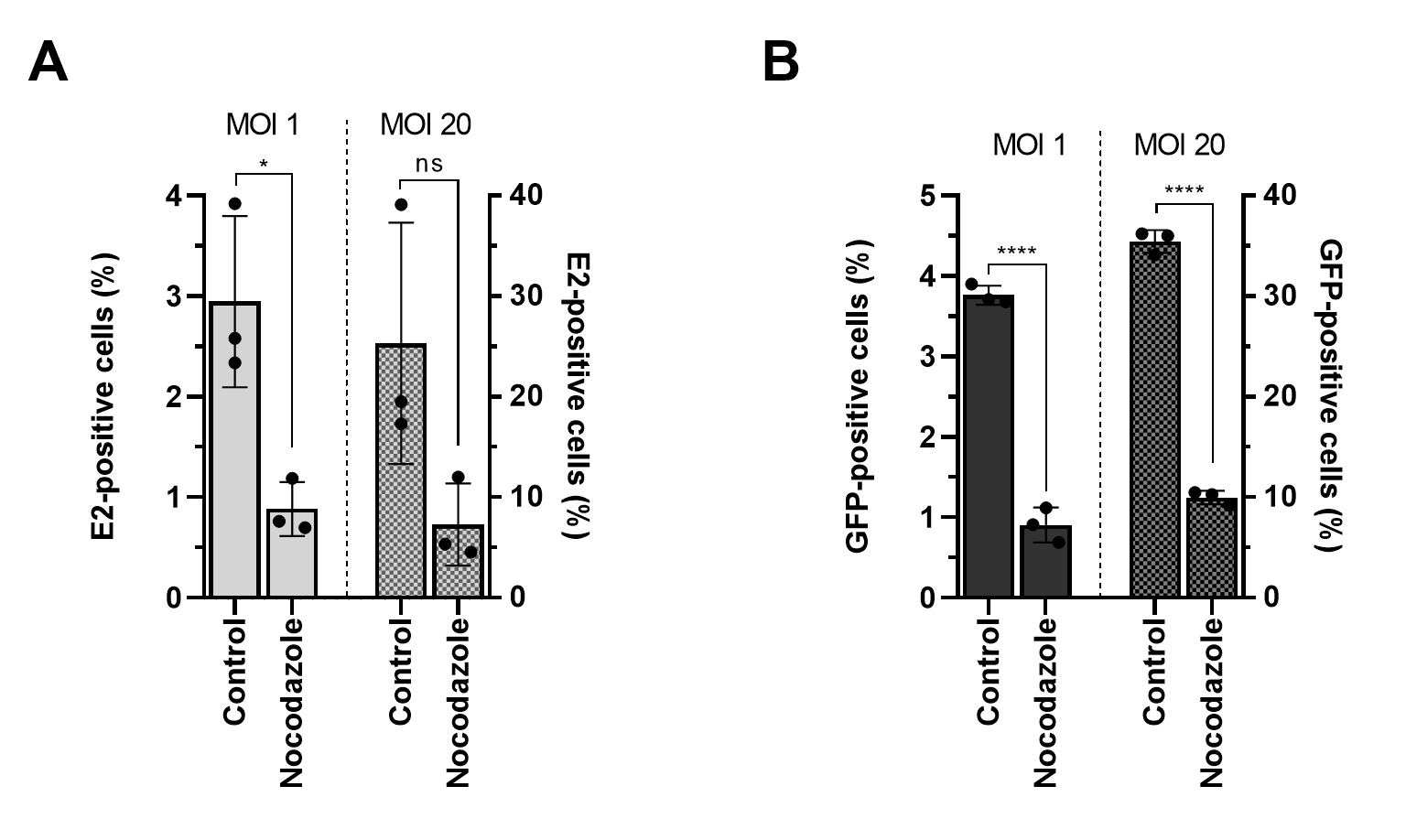

Supplement: S2 Fig — U-2 OS cells were pretreated for 2 h with 10 μM nocodazole and infected with CHIKV LR2006-OPY1 (A) or CHIKV LS3-GFP (B) at MOI 1 or 20 for 10 h. (A) Flow cytometry analysis of E2-positive cells in the presence or absence of nocodazole. (B) Flow cytometry analysis of GFP-positive cells in the presence or absence of nocodazole. Data represents three independent experiments performed in triplicate, each dot represents the average of an individual experiment; error bar represents SD. Data was analyzed and compared to the control using student T-test (*p ≤ 0.05 and ****p≤ 0.0001); ns, not significant. (TIF) [file pntd.0008469.s002.tif]

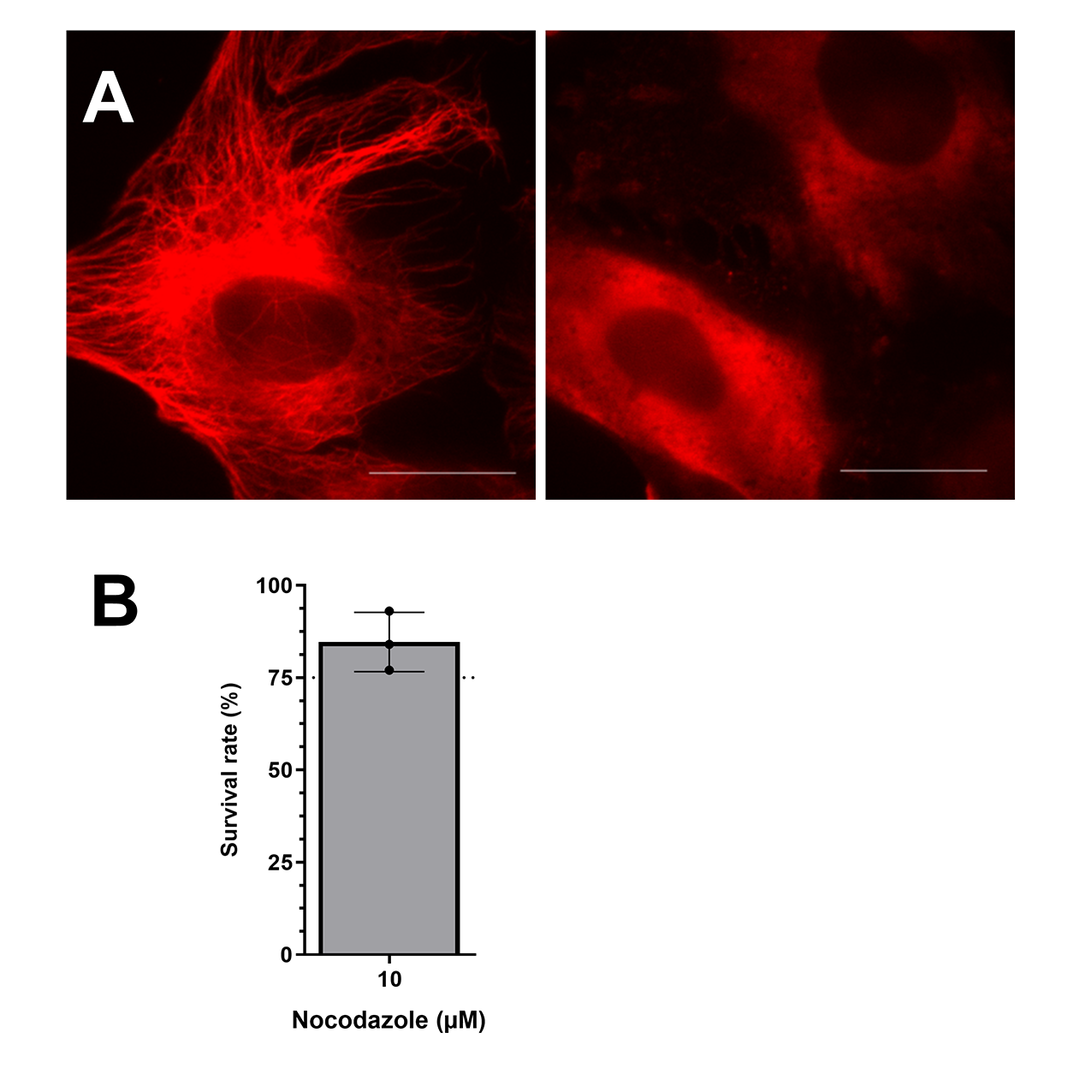

Supplement: S3 Fig — (A) Representative pictures of the α-tubulin staining in BS-C-1 cells in absence (left) and presence (right) of 10 μM nocodazole for 2 h. Scale bar: 25 μm. (B) Cell viability of BS-C-1 cells upon treatment with 10μM nocodazole. The cells were treated for 12h, after which cell viability was assessed using standard ATP lite assay. Dotted line indicates 75% cell survival. Three independent experiments were performed in triplicate; each dot represents the average of a single experiment; error bar represents SD. (TIF) [file pntd.0008469.s003.tif]

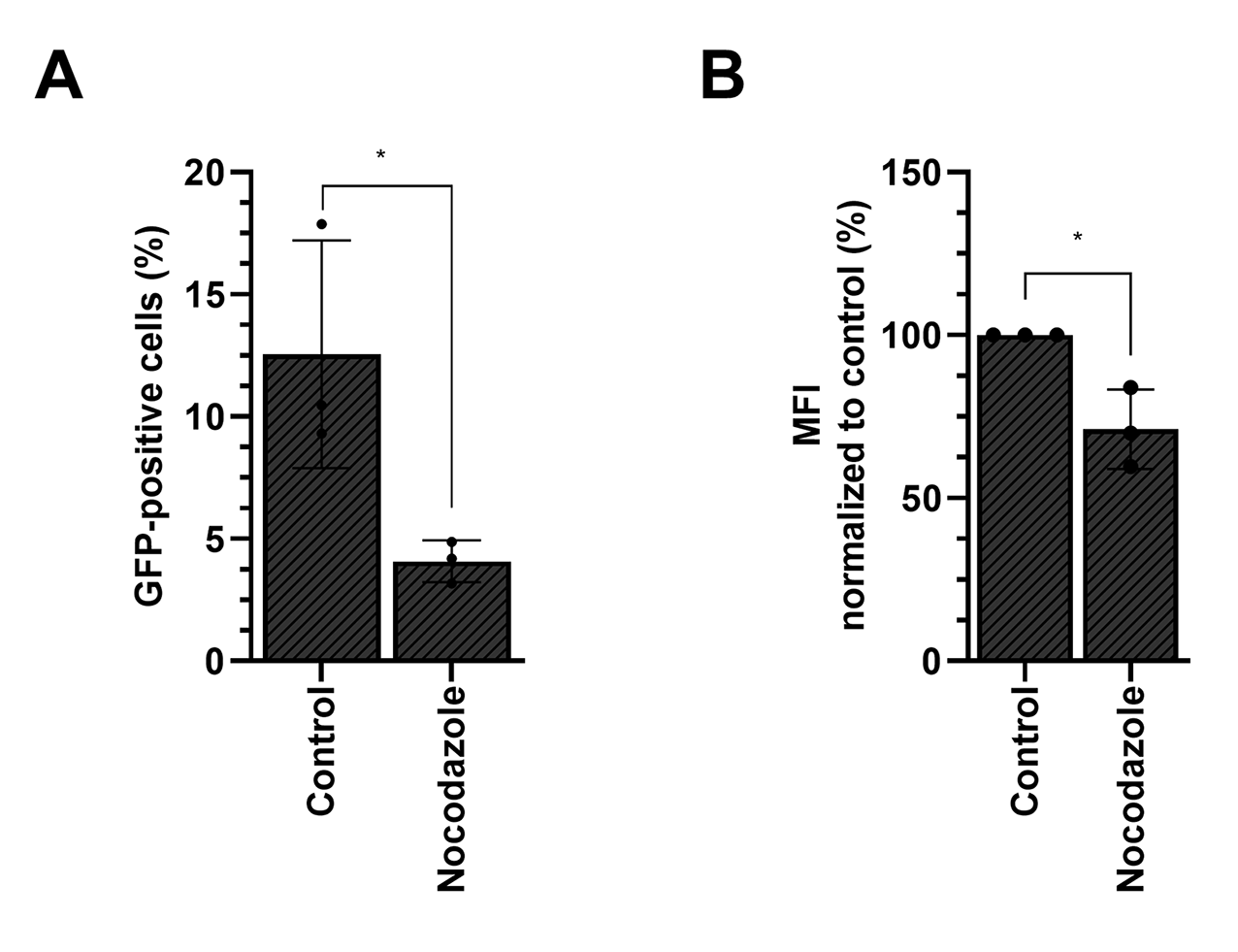

Supplement: S4 Fig — BS-C-1 cells were pretreated for 2 h with 10 μM nocodazole and infected with CHIKV LS3-GFP at MOI 20 for 10 h. (A) Flow cytometry analysis of GFP-positive cells in the presence or absence of nocodazole. (B) Mean fluorescence intensity (MFI) of the infected population. Data is normalized to the positive control. Three independent experiments were performed in triplicate, each dot represents the average of an independent experiment; error bar represents SD. Data was analyzed and compared to the control using student T-test (*p ≤ 0.05). (TIF) [file pntd.0008469.s004.tif]

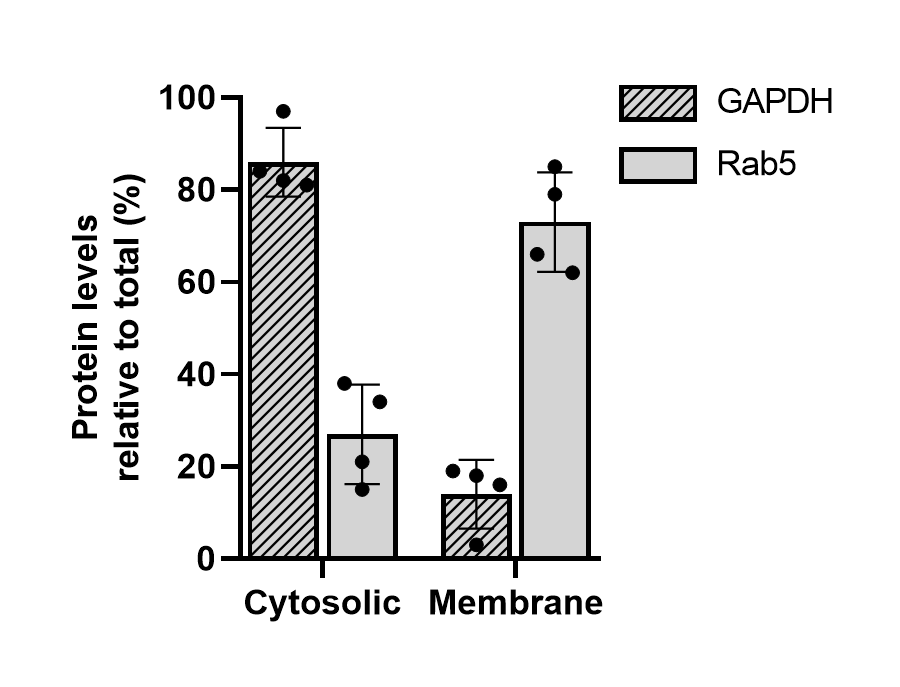

Supplement: S5 Fig — U-2 OS cells were permeabilized using 50μg/ml digitonin for 5 min at RT and subsequently 30 min on ice. Subsequently, the total amount of GAPDH and Rab5 was determined in the cytoplasmic and the membrane fraction by Western blot quantification. Four independent experiments were performed in duplicate, each dot represents the average of an independent experiment; error bar represents SD. (TIF) [file pntd.0008469.s005.tif]
