# Supplementary material for: Chikungunya virus requires an intact microtubule network for efficient viral genome delivery
Source: PLoS Negl Trop Dis. 2020 Aug 7;14(8):e0008469. doi: 10.1371/journal.pntd.0008469 (PMC7413472; doi:10.1371/journal.pntd.0008469)
Supplement: Attachment — Submitted filename: Response to reviewers.pdf [file pntd.0008469.s010.pdf]

Rebuttal

Chikungunya virus requires an intact microtubule network for efficient viral genome delivery

Methods

Reviewer #1: Yes to most questions, other than what's included in my comments below.
No ethical/regulatory concerns.

Reviewer #2: Appropriate. It would have been useful to include an indication of the particle to infectious unit ratio, especially for the DiD labelled virus.

> This is indeed a valid point. We determined this when we developed the DiD-labeling protocol for CHIKV and published the genome containing particle/plaque forming unit-ratios in Hoornweg et al., 2016 (Ref 5 in the reference list of the manuscript, Fig. 1C). Herein, we demonstrated that the GCP/PFU-ratio of DiD-labeled CHIKV is not significantly different compared to wt CHIKV. We have added this reference to the material and methods in line 154.

Results

Reviewer #1: Please see specific comments below

Reviewer #2: The results are for the most part clearly presented. I have just a few questions/comments.

Firstly, the authors show that cell viability is unaffected after 18hrs of nocodazole treatment (Fig S1B), but presumably cell division is inhibited and there are fewer cells in these cultures? If this is the case, does it impact on the data presented in S2B - if there are fewer cells in the nocodazole treated samples, presenting the data as the percentage of infected cells will underestimate the nocodazole inhibition? What are the dotted lines in S1B and S3B?

> Cell viability is assessed with the ATPlite Luminescence Assay System from PerkinElmer. This measures metabolic activity and not the number of cells, though if cells die a lower signal will be obtained. By eye we did not see a drastic difference in cell counts. Importantly, irrespective of the above, the data in Figure S2B shows the percentage of CHIKV-infected cells based on counting 30.000 cells during flow cytometry analysis and therefore is unrelated to the number of cells in culture. Line 217.

> The dotted lines in figure S1B and S3B represents 75% cell viability. We now added this information to the corresponding figure legends (lines 638 & 655).

Why have authors chosen to assess the effects of nocodazole at 10 hrs post infection?
> Growth curve analysis showed that initial CHIKV particle production is seen at 8 hpi and continues to increase thereafter. We chose 10 hpi as this increases the sensitivity yet in the used assays still reflects 1 round of replication. Line 268. The LS3 infection kinetics are described in detail by Scholte et al. (2013 + 2015) and confirmed experimentally in our lab (data not shown). Comparable date was obtained for CHIKV-LR OPY1 strain (Bouma et al., 2020).

Using DiD as a fusion reporter does detect hemifusion, but it also reports full fusion. In these assays the authors cannot distinguish between hemifusion and full fusion. So I think it misleading to use hemifusion throughout the paper, and I suggest they use 'hemifusion/full fusion' instead.
> We agree with the reviewer that this can be misleading. Yet we do not know if the hemifusion intermediate always proceeds to full fusion. We now tried to explain the terminology more clearly in line 180-184

Can the authors be certain that the fast directed movement described in Fig 2A is intracellular and not on the cell surface? I.e. could it be viral surfing?
> In order to assess this question, we investigated fast-directed movement in cells transfected with Clathrin(-YFP) or the early endosome marker Rab5(-GFP). The results (shown in Fig. 3D) show that this type of movement is seen <u>after</u> the process of clathrin-mediated endocytosis and prior to arrival to Rab5-positive early endosomes. Mostly likely transport occurs directly after the clathrin coat dissociates from the intracellular vesicle. Therefore, we conclude this type of movement occurs intracellular and is not viral surfing.

Line 291 - there does seem to be a 30% change in MFI.
> The reviewer is correct and we adjusted the text accordingly. Line 293.

Figs 2A and B - the labeling of the boxes is defective.
> We have adjusted the labeling in these figures.

Line 375 - how do the authors define perinuclear? The two events illustrated in Fig 2A do not appear to be perinuclear, yet in Fig 3/Line 398 the authors give a quantitative analysis of perinuclear v non-perinuclear fusion.
> Correct, both particles fused in the periphery. The location of fusion is determined by eye. When a virion fuses closer to the nucleus than the plasma membrane fusion was determined as perinuclear. Vice versa, when the particle was closer to the plasma membrane than the nucleus fusion was determined as periphery. The image shown in Figure 2a was primarily selected for visual purposes, as it contains multiple trajectories in which fast directed movement was observed. By chance, the fusion events of both trajectories occurred in the periphery. Line 171/172.

Conclusions

Reviewer #1: Yes to all.

Reviewer #2: The conclusions are appropriate for the data presented. However, I am concerned that the authors do not cite and discuss relevant published work appropriately. Specifically, papers by Vonderheit and Helenius (PLoS Biol 2005 e233) describing morphological analysis of Semliki Forest virus is not mentioned; and though cited, Bernard et al's. report of clathrin-independent internalization of CHIKV into cells is not discussed.

> In the paper of Vonderheit and Helenius, SFV E1 and E2 were coupled to different fluorophores, and subsequently used as a cargo to study endosome sorting and trafficking. In their paper, Vonderheit and Helenius found that microtubules are likely involved in the separation of (SFV-carrying) early endosomes and Rab7-positive carrier vesicles. As the observed action of microtubules described by Vonderheit and Helenius occurred after escape of the virus from the endosome, their results are very relevant to endosome sorting, but not to SFV or CHIKV infection. Therefore, we did not discuss this data in our paper. Furthermore, upon microtubule disruption no effect on SFV infection was observed, which the authors explained by the fact that SFV escapes from the early endosome. This might be relevant for our study, however their results are difficult to assess because the authors do not show the data and it is unclear from the Material and Methods whether nocodazole treatment was present during SFV infection. We know that nocodazole treatment is reversible and therefore it is crucial that nocodazole is present during viral infection. For these reasons we did not include this manuscript in the reference list.

> Findings described in the paper from Bernard et al., 2010 are indeed discussed in lines 524-529. We did, however not discuss the part on clathrin-independent internalization in this paper as we have shown that in BS-C-1 cells CHIKV entry occurs primarily via CME (Hoornweg et al., 2016).

Editorial and Data Presentation Modifications

Reviewer #1: Please see specific comments below

Reviewer #2: The paper should be carefully edited for word usage, etc.

Summary and General Comments

Use this section to provide overall comments, discuss strengths/weaknesses of the study, novelty, significance, general execution and scholarship. You may also include additional comments for the author, including concerns about dual publication, research ethics, or publication ethics. If requesting major revision, please articulate the new experiments that are needed.

Reviewer #1: In this manuscript, Hoornweg et al elucidate the role of the microtubule network in the trafficking of CHIKV particles early in the course of infection. They hypothesize that microtubules may direct the particle to a cellular location that is beneficial for establishing infection or aids in nucleocapsid uncoating. They monitored trafficking of DiD-labelled viral particles and observed two

distinct patterns prior to membrane fusion in the early endosomes. Using nocodazole to disrupt the microtubule network, they observed reduced number of infected cells, restriction of fusion to the cell periphery, and impaired delivery of the viral genome into the cytoplasm.

The microtubule network has been previously implicated in the entry of multiple other viruses, and thus the innovation here is limited to the demonstration of this requirement for CHIKV and the discovery of the two distinct trafficking patterns. While the data demonstrating the two distinct viral populations is interesting, it remains unclear what is the functional relevance of this difference and what is the mechanistic role mediated by microtubules in CHIKV entry.

Major comments:

1. The magnitude of the effect shown in most figures is small (less than a 2 fold difference in many cases – e.g. Fig. 1A, Fig. 4B), which makes it unclear what the biological relevance of the findings is.
> Fig. 1A shows 70-80% reduction in infectivity. In 4B we used shorter incubation times which might slightly reduce the observed effect. In our view given the sensitivity of the applied assays a 2-fold reduction in infectivity is biologically relevant.

2. The number of infected cells is very low (2.9% in an MOI of 1, 9.2% in an MOI of 20 as per line 288). Why did the authors choose to infect the cells only for 30 min (when a more standard infection time is 1 hour for alphaviruses)? Perhaps a longer infection time will increase infection rate and improve the dynamic range?
> In standard infectivity experiments the incubation time is 1.5 hrs (see line 211). However in case of indicated experiments we have shortened it because our previous paper (Hoornweg et al., 2015) demonstrated that fusion events occur very rapidly after addition of the virus to cells.

3. The authors should demonstrate a dose-response effect for the various phenotypes shown with nocodazole treatment.
> We do not fully understand the remark of the reviewer. We used a non-toxic nocodazole concentration that completely disrupted the microtubule network. At lower concentrations, the microtubule network is at least partially intact and it is hard to draw conclusions from this. For similar reasons, single virus tracking was only done at the concentration were the microtubule network is disrupted.

4. Is it possible the cell cycle arrest at G2/M induced by nocodazole accounts for the observed reduction in infection and trafficking of viral particles?
> Nocodazole is indeed known to be used in cell biology to synchronize the cell division cycle as cells arrest at G2/M-phase when treating for a prolonged time (>12-16h). This arrest is a direct consequence of microtubule disruption, as the cell cannot assemble the mitotic spindle.
In most experiments we treat the cells for 2h prior to infection. Tracking experiments are recorded within 30 min. Therefore, there is no reason to believe that the altered trafficking behavior is an indirect consequence of cell cycle arrest but rather a direct effect of the disruption of the microtubule network.

5. The distance and velocity traveled by individual viral particles needs to be shown in figures 2 and 3.
> Unfortunately, due to cropping of the movies for proper trajectory analysis, the original dimensions of the images analyzed were not recognized by the analysis software. Therefore, the exact velocity of the particles is unknown and the distance travelled by these particles cannot be calculated. Consequently, the relatively velocity over time as reported by the analysis software is shown in figures 2 and 3. Although the exact velocity cannot be given, these graphs, together with the provided images and scale bars, show that during the burst of directed movement the viral particles shown in Fig. 2a travel approximately 2,5-3,5 µm in 4-5 seconds. From this, one can calculate a velocity of 0,5-0,8 µm/s during directed movement, which is in line with the velocity reported for microtubule-dependent movement in literature (on average 0,3-1,5 µm/s (Kulic et al., 2008)).

6. In Fig. 4A, the Western blot should include all 3 conditions (control, and two treatments).
> The Western blot is performed to confirm cell fractionation in each experiment. To detect this, we add an additional well to each experiment (purely for WB). As the fractionation protocol is independent of the treatment condition we did not perform WB for all three treatment conditions. In our view this is not necessary and due to the lock-down of our lab due to COVID19 we are unable to perform these experiments at this moment in time.

Additional comments:
1. Graphs in all figures – would be better to show controls in each figure rather than showing just the % (or alike) difference relative to control.
> We believe this is a matter of taste. We prefer to show % inhibition but will change it if the above is the policy of the journal.

2. Would be good to avoid single column graphs (as in 1D). These can be just mention in the text.
> We do think these graphs are informative as they show the experimental replicates and standard deviations, therefore we for now did not remove this data from the figures.

3. Figure 1E – a schematic showing time of drug addition would be helpful.
Thank you, added

4. Figure 3D – the legend should explain what before, during and after means.
Thank you, added

5. Embedding figure legends in the text makes it harder to review.
We followed the guide lines given by PLOS Neglected Tropical Diseases, as they stated “Insert figure captions in manuscript text, immediately following the paragraph where the figure is first cited (read order). Don’t include captions as part of the figure files themselves or submit them in a separate document”.

Reviewer #2: Overall, this work provides a modest step forward in understanding CHIKV entry into cells. Unfortunately, there is really no indication of how microtubule based transport of CHIKV favors penetration and infection.

This report describes for the first time that CHIKV particles traffic along microtubules during cell entry and that gRNA release is impaired in nocodazole-treated cells. The exact role of nocodazole is indeed not uncovered and this is primarily due to the fact there are no methods available to capture the transient and dynamic events of CHIKV nucleocapsid delivery/uncoating.
